# Exploring the Potential of Chemically Matched Fragments as Internal Standards for Quantitative SERS with Panobinostat

**DOI:** 10.1021/acs.analchem.5c02017

**Published:** 2025-08-21

**Authors:** Yiming Huang, Yikai Xu, Chunchun Li, Steven E. J. Bell

**Affiliations:** † School of Chemistry and Chemical Engineering, 1596 Queen’s University, David Keir Building, Stranmillis Road, Belfast BT9 5AG, U.K.; ‡ Key Laboratory for Advanced Materials and Feringa Nobel Prize Scientist Joint Research Center, Frontiers Science Center for Materiobiology and Dynamic Chemistry, School of Chemistry and Molecular Engineering, 47860 East China University of Science and Technology, 130 Meilong Road, Shanghai 200237, China; § School of Materials Science and Engineering, 47860 East China University of Science and Technology, 130 Meilong Road, Shanghai 200237, China

## Abstract

Surface-enhanced Raman spectroscopy (SERS) has great potential for therapeutic drug monitoring (TDM) due to its high sensitivity; however, achieving accurate and robust quantitative data remains challenging. The most effective approach for quantitative SERS is to use internal standards (IS). Isotopologues are particularly effective; however, these or closely related analogues of the target compound are often unavailable. We have addressed this problem by using fragments of the target compound as the IS. Here, 2-methylindole (2-MI) was identified as the most suitable fragment for Panobinostat (Pano). Tests with an enhancing colloid that degraded significantly over a 4-week period showed that the 2-MI IS allowed changes in the absolute intensity of the Pano signal over time to be corrected and that the standard error achieved with the degrading enhancing medium matched that of results obtained with the fresh colloid. The calibration range could be extended by using either low (4 × 10^–7^ M) or high (10^–5^ M) concentrations of 2-MI, both of which gave linear log/log Pano calibration curves (*R* ^2^ = 0.993 and 0.998, respectively) although these curves had different slopes. In contrast, thiophenol (TP), a nonchemically matched IS, gave reasonable results at a low (10^–6^ M) concentration (*R* ^2^ = 0.942) but completely failed at a high (10^–5^ M) concentration due to surface saturation. These findings can be rationalized using a model where, at submonolayer concentrations, both the target and IS adsorb to the nanoparticle surface in proportion to their concentrations; however, at higher concentrations, competition for active sites on surface alters the relative intensities observed. This fragment-based approach significantly increases the availability of chemically matched IS for large target molecules and therefore significantly broadens the range of compounds where robust quantitative SERS analysis is possible.

## Graphical abstract

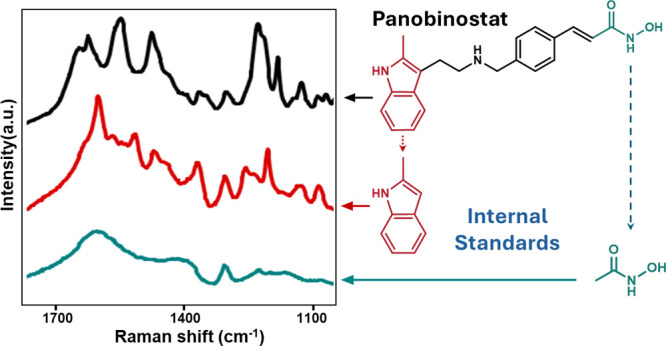

## Introduction

Therapeutic drug monitoring (TDM) is a pivotal component of contemporary clinical practice, ensuring the optimization of drug therapy for patients.^1^ Classes of therapeutic drugs that particularly benefit from TDM are those whose therapeutic concentrations are close to their toxic concentrations, such as anticancer drugs.^2,3^ Most routinely used analytical methods in TDM rely on immunoassays or separation techniques combined with mass spectrometry (MS).^1^ While immunoassays are user-friendly, they may yield false negative or positive results due to nonspecific adsorption.^4^ In contrast, liquid chromatography (LC) coupled with MS offers a robust platform for anticancer drug quantification, addressing some of the challenges posed by immunoassays. However, it is time-consuming, requires sophisticated analytical laboratory settings and is expensive.

Surface-enhanced Raman spectroscopy (SERS),^5^ has emerged as a powerful but nondestructive analytical technique offering distinct advantages compared to the currently used techniques for TDM.^6,7^ SERS utilizes localized surface plasmon resonance (LSPR), typically generated on the surface of noble metal nanoparticles (NPs), to address the challenges of low sensitivity and fluorescence interference associated with conventional Raman scattering.^8,9^ This LSPR effect amplifies Raman scattering from molecules positioned near the surface, particularly accentuated when analyte molecules reside within the overlapping plasmons of closely packed nanostructures, often termed plasmonic “hot spots”.^10^

Notably, the sensitivity of SERS can reach detection levels in the subnanomolar range even in complex biological matrices, providing limits of detection and quantification commensurate with those encountered in therapeutic applications.^1,11,12^ Additionally, the characteristic Raman scattering enables molecular fingerprint identification and the simultaneous multiplex detection of several analytes by SERS.^13,14^ This is of paramount importance for TDM, given that patients are often treated with multiple anticancer drugs concurrently.^15–19^ Furthermore, SERS spectroscopic investigations can be conducted in the presence of water, allowing for the application of these techniques in the investigation of organic and biochemical substances in their natural environment.^20,21^ Finally, the availability of relatively low-cost hand-held systems means that bedside measurements may eventually be possible, provided the issues discussed below are overcome.^22^

While SERS has numerous advantages, making quantitative SERS measurements is challenging. This is principally because the absolute intensity of the SERS signals depends not only on the concentration of the target analyte but also on a large number of experimental factors which are difficult to control. Some of these factors such as variations in laser power or focus position can be eliminated by engineering controls of various types.^23,24^ The more challenging problems are associated with changes in the enhancement factor provided by the SERS substrates.^25^ These are particularly notable for metal colloids where the plasmonically active “hot spots” are created by aggregating the colloid. Variations in the extent of aggregation will result in significant changes to the enhancement factor and, therefore, the SERS signal, even if the concentration is unchanged.^26^ In addition, any changes to the experimental conditions which change the affinity of the target molecules for the surface will also change the number of molecules in the enhancing regions and therefore the intensity of the SERS signal. Numerous experimental factors can interfere with the binding of the target either by physically blocking access to the surface (as is often observed with proteins, for example) or occupying surface sites so they are no longer available.^27,28^ In addition, the surface charge may change or the enhancing media may degrade over time due to oxidation or irreversible aggregation and precipitation of the NPs in metal colloids.^29,30^

Incorporating an internal standard (IS) is a widely accepted and effective method for achieving robust calibrations in analytical chemistry that are stable against changes in experimental conditions. It is particularly useful in SERS where the external perturbations can lead to very large changes in signal. In principle this should be straightforward, a standard SERS active material is added to the system and the signals of the target molecule are normalized against the intensity of the standard. If the experimental conditions change, the signals of both standard and analyte will vary in the same way so normalization will remove the effect of the change. The simplest IS are strongly scattering compounds with high affinity for the surface.^31,32^ These need to be added at low concentration because they will block surface sites and therefore reduce the signal from the analyte. An elegant solution to the problem of surface site blocking is to introduce the IS as an internal layer in core–shell particles whose exterior surface is therefore left free for interaction with the target.^33–35^ In both these cases, the IS can compensate for changes in the signal due to physical factors which affect the signals, such as the number of particles in the probe beam or changes in the plasmonic properties of the enhancing substrates. However, they are not able to compensate for chemical factors that alter the binding of the analyte to the enhancing surface, since their signals will remain constant even under conditions where the binding changes. In contrast, differences in binding can be compensated for by using isotopologues of the analyte as the IS because both analyte and its isotopologues are expected to respond identically to all changes in the experimental conditions, including chemical changes to the enhancing surface, plasmonic effects and simple physical variations such as focus position.^36,37^ This approach has been shown to be extremely effective for a broad range of analytes and is notable as the basis for a commercial assay used to quantify monoethanolamine, where the calibration using the ratio of peaks from monoethanolamine with its *d*_4_-isotopologues was shown to be stable over 4.5 years and 25 instruments.^38^

Unfortunately, the isotopologue-based approach is hindered by the low availability and high cost of suitable isotopologues. An alternative approach that seeks to retain the advantages of isotopologue-based IS while also avoiding these problems is to use “chemically matched” IS, which are more readily available compounds that are selected on the basis of having similar chemical properties to the analyte, in particular in their binding to the enhancing surface.^39^ This approach has been used for quantitative analysis of nicotine, where pyridine was used as the chemically matched standard^40,41^ and for 4,4′-methylenedianiline, where 3,3′-methylenedianiline was used.^42^

Commonly, however, for large molecules with complex structures there are no closely related molecules suitable for use as IS. For example, there are no close analogues of the Pano target analyte used in this study. However, Pano can be readily divided into several easily identified chemical fragments and here we introduce the concept of using these fragments, as chemically matched IS. In this approach appropriate fragments of the target molecule are each tested separately to determine how they bind to the enhancing surface and therefore which will be the most suitable as IS.

Here it was found that 2-methylindole (2-MI) was suitable for use as a chemically matched fragment IS for quantitative detection of Pano. The use of the 2-MI was shown to significantly improve the stability and robustness of quantitative SERS measurements with Au colloid over time scales of weeks. Furthermore, we also show that the use of chemically matched IS could dramatically increase the detection range to concentrations well above those needed for monolayer coverage. This is not possible for preadsorbed IS, whose signal plateaus at monolayer coverage, but it can be observed with chemically matched IS, as the mechanism switches from filling vacant sites at low concentration to competition between analyte and IS at higher concentration. In the current study, calibration plots above and below monolayer coverage, with *R* ^2^ > 0.99, were determined, but the slope of each was different, reflecting the change in adsorption mechanism in the two ranges. With a preadsorbed strongly binding thiophenol (TP) standard, the submonolayer calibration was also good (*R* ^2^ > 0.94), but it completely failed at the higher concentration range. These findings are crucial to fully harnessing the potential of SERS in TDM and also extend to broader practical applications of SERS in other scenarios.

## Experimental Section

### Chemical Reagents

The anticancer drug, Panobinostat, was purchased from MedChemExpress LLC. All its chemical fragments, 2-methylindole, 4-methylbenzylamine, acrylamide, tryptamine, acetohydroxamic acid, and pyrrole were purchased from Sigma-Aldrich Ltd. Panobinostat and all its chemical fragments were dissolved and diluted to different concentrations in ethanol for consistency. The water used in all experiments was low TOC (<3.0 ppb) 18.2 MΩ cm^–1^ distilled deionized (DDI) water.

### Preparation of Citrate-Reduced Silver Colloid (CRSC)

CRSC was synthesized according to the method reported by Lee and Meisel.^43^ 0.045 g silver nitrate (AgNO_3_) was weighed and dissolved in 250 mL DDI water and brought to boil under reflux and vigorous stirring. Meanwhile, 0.100 g trisodium citrate (Na_3_C_6_H_5_O_7_) was dissolved in 10 mL DDI water to obtain a 1 wt % solution. As soon as the mixture solution began to boil, 5 mL of the 1% trisodium citrate aqueous solution was added dropwise into the boiling solution with a syringe over 30 s. After the addition of sodium citrate, the reaction mixture changed from colorless to light yellow and finally to cloudy green within 15 min, indicating the successful formation of Ag NPs. The colloid was heated for another hour to allow the reagents to completely react before being cooled down to room temperature.

### Preparation of Citrate-Reduced Gold Colloid (CRGC)

CRGC was prepared following the method presented by Frens with slight modifications.^44^ In brief, 50 mL of HAuCl_4_·3H_2_O aqueous solution containing 0.05 g of HAuCl_4_·3H_2_O powder was heated with stirring under reflux. As soon as the solution began to boil, 5.6 mL of 1% (w/w) trisodium citrate aqueous solution was directly added into the HAuCl_4_·3H_2_O solution by syringe all at once. The color of the resulting mixture quickly turned from transparent pale yellow to wine red, which indicated the formation of Au colloid. The colloid was allowed to react for another 15 min before being cooled down to room temperature. The AuNPs synthesized had an average diameter of ∼55 ± 15 nm. The CRGC prepared had a zeta potential of ca. −40 mV and a particle concentration of ∼2 × 10^11^ particles/mL. The UV–vis spectrum of the prepared CRGC shows a single extinction peak at 540 nm. Upon addition of the aggregating agent (NH_4_)_2_SO_4_, the peak significantly broadened and decreased in intensity, indicating the formation of aggregates (Figure S1).

### SERS Measurements

SERS spectra in this work were all acquired on an Avalon R2 Raman station equipped with a 785 nm laser with 30 mW laser power. The total accumulation time for each SERS spectrum was 30 s. To obtain SERS spectra of blank CRSC and CRGC, 200 μL of metal colloid was mixed with 20 μL of ethanol and aggregated by 1 M aggregating salt ((NH_4_)_2_SO_4_ was used in this work) within a well of a polypropylene 96-well plate. For simple Pano detection by SERS, 200 μL of metal colloid was mixed with 20 μL of drug solution and aggregated by 1 M (NH_4_)_2_SO_4_ solution within a well of a polypropylene 96-well plate. For SERS detection of Pano with various IS, equal amounts of various concentrations of Pano were mixed with a fixed concentration of IS to create multiple mixture solutions with different ratios of Pano to IS. 200 μL of metal colloid was mixed with 20 μL of Pano/IS mixture solution and aggregated by 1 M (NH_4_)_2_SO_4_ solution within a well of a polypropylene 96-well plate for simultaneous SERS detection of both components. The measurements of relative intensity ratios were performed by importing spectral data files into Microsoft Excel and logarithmic transformations on the values of intensity, concentration and intensity ratio were performed as needed.

## Results and Discussion

### SERS Detection of Pano and Its Fragments

Pano has not previously been analyzed using SERS. The SERS spectra of Pano on Ag and Au colloid are compared with the normal Raman spectrum in Figure S2. The SERS spectrum on Ag has major peaks at 1638, 1608, 1230, and 1186 cm^–1^ (Figure 1B­(a)) which are close to those of the normal Raman spectrum although there are some changes in relative intensity. Surprisingly, the SERS spectrum on Au is very different since, as well as these features, it contains several additional strong bands (Figure 1C­(a)). This immediately suggest that Pano interacts differently with the surfaces of the Ag and Au enhancing NPs, although it does adsorb to both. In order to understand the reasons for these differences, the Pano was divided into several fragments which could be modeled using smaller and easy to obtain compounds that might also potentially be used as IS.

**Figure 1:**
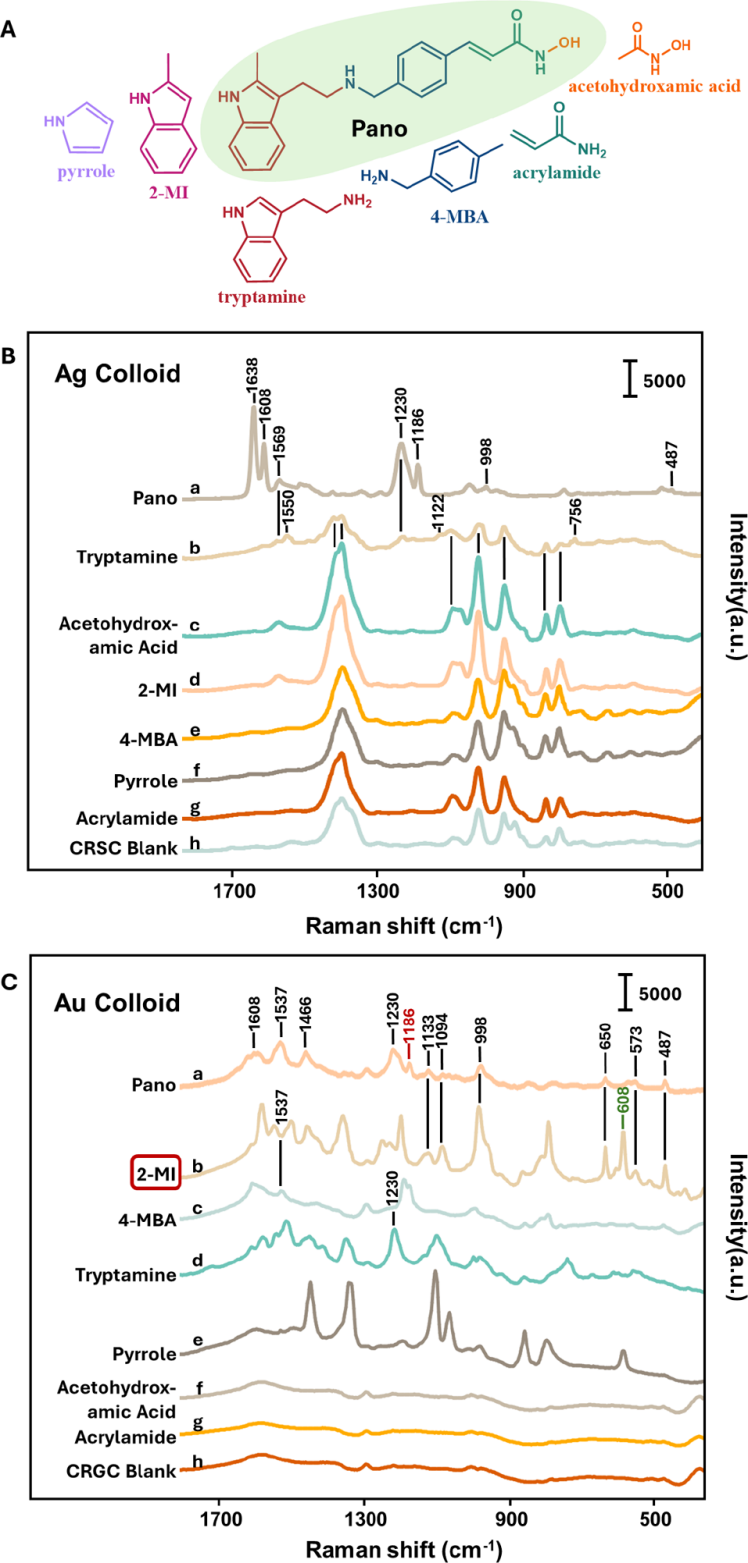
(A) Chemical structures of Pano and its chemical fragments detected within this experiment. SERS spectra of 10^–5^ M of Pano, 2-MI, 4-MBA, acrylamide, tryptamine, acetohydroxamic acid, and pyrrole on Ag (B) and Au (C) along with blank Ag and Au SERS spectra. 2-MI is highlighted due to its high spectral similarity with Pano. Marker bands for Pano at 1186 cm^–1^ and for 2-MI at 608 cm^–1^ for following analysis are highlighted. The SERS spectra were offset vertically for clarity.

The process is illustrated in Figure 1A, which includes the structures of the model fragments chosen: 2-methylindole (2-MI), 4-methylbenzylamine (4-MBA), acrylamide, tryptamine, acetohydroxamic acid, and pyrrole. The combination of tryptamine, 4-MBA, acrylamide, and acetohydroxamic acid can form a complete Pano structure. Although the chemical structure of 2-MI is similar to that of tryptamine, both were included since they allow the effect of the additional NH_2_ group to be examined. Pyrrole was included because it is a commonly encountered molecule with a thoroughly studied structure, which aids in the analysis.

The SERS spectra of Pano and its fragments on Ag and Au are illustrated in Figure 1B,C, respectively. Notably, on Ag most of the fragments do not show any strong characteristic bands and the spectra are dominated by the citrate signals that are also observed in the blank spectrum. The only exception is tryptamine (Figure 1B­(b)) which in addition to the citrate bands has weak features at 1569, 1550, 1230, and 756 cm^–1^. This suggests that the adsorption of Pano onto the Ag surface is not due to a strong interaction from any individual functional group but arises from a cooperative effect, where the weak interactions from various functional groups in the drug combine.

In contrast to Ag, the SERS spectra show that most fragments can adsorb to the Au surface and produce distinct peaks that correlate with those of Pano. However, acetohydroxamic acid and acrylamide (Figure 1C­(f,g)) show spectra identical to the CRGC blank, suggesting they cannot be detected using Au as the SERS substrate. However, 2-MI, 4-MBA, tryptamine and pyrrole all give rich spectra with numerous strong bands (listed in Table S1). All of the aromatic fragments tested could potentially be used as IS, but it is best to use the one that is most chemically similar to the Pano target, meaning the one that binds in a similar way in this context. Although there is no simple method for rigorously characterizing the detailed nature of the interactions between the molecules and the enhancing surface, it is possible to assess which fragments absorb in the same way as Pano by comparing their SERS features. In this case, the 2-MI and tryptamine spectra are both similar to that of the drug, suggesting that the drug binds to Au through its indole ring. However, 2-MI was more suitable as an IS since its low frequency bands are larger and are convenient for quantitative measurements. It is important to note that the differences between the spectra of Pano and 2-MI are not due to differences in adsorption behavior but instead reflect the fact that they are differently substituted indole derivatives, in much the same way as is observed in Figure 1C for tryptamine and 2-MI.

We assigned the observed Pano peaks by referring to literature data on structurally relevant fragment molecules (with chemical structures shown in Figure S3).^45–48^ The Pano peaks observed at 487, 573, 1133, 1230, 1466, and 1537 cm^–1^ are attributed to characteristic vibrational modes of the indole ring. In particular, the peak at 992 cm^–1^ corresponds well to the out-of-phase breathing mode of the indole ring. The peak at 1186 cm^–1^, which is used for the following quantitative analysis of Pano and also observed in 4-MBA (Figure 1C, a and c), is likely due to C–H in-plane deformation or C–N stretching. Additionally, the peak at 608 cm^–1^, employed as a marker for 2-MI quantification, aligns with a similar feature in the SERS spectrum of pyrrole (Figure 1C, b and e), suggesting that this vibrational mode can be assigned to the pyrrole moiety present within the indole framework.

### Quantitative Measurements of Pano and IS Calibration

Initial quantitative measurements of Pano were conducted on both Ag and Au surfaces without any IS. The SERS spectra of Pano on Ag colloid recorded at a series of concentrations showed the intensity of the 1186 cm^–1^ peak in a log/log plot grew linearly over the range 10^–8^–4 × 10^–7^ M (*R* ^2^ = 0.987) and then plateaued, barely changing intensity up to 10^–5^ M (Figure S5b). Similarly, with Au, measurements of the 1186 cm^–1^ peak in the SERS spectra of Pano over the concentration range 10^–7^–10^–5^ M (Figure 2A) showed a linear log/log response between 10^–7^ and 2 × 10^–6^ M, followed by a plateau region (Figure 2B, W0). In this case the *R* ^2^ value of the linear section was 0.988, while the standard error in the slope was 0.05. These calibrations are sufficiently good for most purposes, however, they were obtained using the same batch of colloid on the same day, while for practical purposes it is important that the calibration is stable over reasonable time scales of days to weeks, so the experiments on Au were repeated using the same colloid after 2 and 4 weeks. The results of these measurements are also shown in Figure 2B as W2 and W4. In these plots, although the slopes of the calibration lines remain similar, the *R* ^2^ values decrease to 0.980 for W2 and to 0.939 for W4. More importantly, there is also a significant decrease in the absolute intensities of the signals, which suggests that there is some degradation in the plasmonic properties of the enhancing colloid over time. Clearly, measurements conducted at later times that used the original calibration to obtain the sample concentration would be extremely inaccurate, and for reliable results the calibration would need to be repeated frequently to compensate for the changes with time.

**Figure 2:**
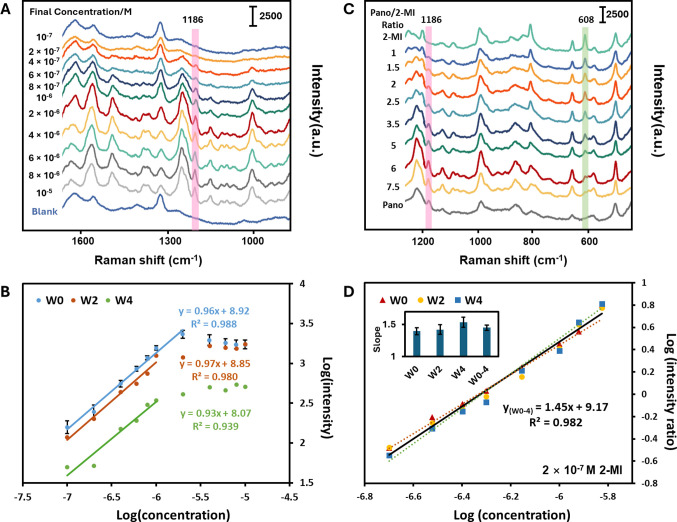
(A) SERS spectra of a series of concentrations (indicated with each line in figure) of Pano detected on Au. The band at 1186 cm^–1^ which was used in the calibration is highlighted. (B) Calibration curves for SERS signal intensity (1186 cm^–1^) versus Pano concentration on Au for 3 different sets of measurements (11 concentrations each set) taken at 2-week intervals (week zero to week four, W0–W4). Error bars for the W0 data are calculated from 5 replicate experiments using the same batch of colloid conducted on the same day. (C) SERS spectra of a series of Pano/2-MI mixtures with different ratios detected on Au. Characteristic bands for Pano (1186 cm^–1^) and 2-MI (608 cm^–1^) are marked. (D) Plots of the logarithmic concentration of Pano against the logarithmic intensity ratio of Pano/2-MI characteristic bands (1186:608 cm^–1^) on Au for 3 sets of measurements (8 ratios each set) taken at 2-week intervals (W0–W4). Solid line is linear regression fit to all the data points recorded W0–W4, and dotted lines show the standard error range in the slope. The inset bar chart shows the linear regression slopes for W0, W2, and W4, respectively, in comparison to the overall W0–W4 data set. The concentration of 2-MI used was 2 × 10^–7^ M.

The alternative to frequent recalibration is to use an IS, preferably one that is chemically matched so that its signal changes in the same way as that of the target analyte as the substrate degrades over time. Here, we used 2-MI as a chemically matched fragment IS, for the reasons discussed above. Although the spectra of the 2-MI and Pano are similar, there are well-differentiated unique bands in the target (1186 cm^–1^) and IS (608 cm^–1^) spectra which allow the relative intensities of the target and IS to be ratioed. The main reason for choosing these bands is to avoid overlap between features from both species. The nature of the vibrational modes giving the bands is not critically important for the quantitative analysis since the relative intensities of all the bands characteristic of each compound remain unchanged with concentration (as shown in Figure S4) and therefore any of them could be selected, provided they meet the other requirements discussed above.

Similar to other IS methods, the quantitative SERS detection in this study simply involved mixing the samples of interest with the chemically matched IS at a fixed concentration before incubating the mixture with a colloidal solution. Following SERS acquisition, the concentration ratio of the analyte and the IS was determined from the ratio of the spectral features associated with the two compounds. The first step was to establish the adsorption characteristics of the 2-MI, since this is useful in selecting appropriate concentration of 2-MI to use as an IS. Figure S6 shows a log/log calibration plot for the 608 cm^–1^ of 2-MI on Au, which is linear over the range 10^–7^–10^–5^ M. The similarity of this concentration range to that of Pano on Au (10^–7^–2 × 10^–6^ M), along with the similarity of the slopes of the calibration lines (0.72 vs 0.96, i.e., differing by <25%), indicates that the Pano and 2-MI have similar adsorption properties to the Au surface, and therefore that the 2-MI is, as expected, chemically matched to the Pano.

Figure 2C illustrates the SERS spectra of Pano and 2-MI mixtures at different ratios, alongside the pure spectra of each compound. The relative sizes of the characteristic SERS spectral features of Pano (1186 cm^–1^) and for 2-MI (608 cm^–1^) change gradually with different Pano/2-MI ratios in the expected way. Calibration curves were established by plotting the log peak height ratio against the log Pano concentration, measured on 3 different days at approximately 2-week intervals, as shown in Figure 2D. The calibration curves using the IS are significantly more consistent than those without (Figure 2B), as shown by the overlap of the points recorded on different weeks in the plot. A more quantitative assessment of the effect of recording spectra over a long-time range is to compare the calibration plot which includes data from all the time points with that from W0. In this case, although the slope for the extended time range data plot W0–W4 was slightly different from that recorded on W0 (1.45 vs 1.39), the standard error in the slope was lower for the extended data set (0.04 vs 0.05). This means that the calibration was actually slightly better for the entire set of data recorded over the extended time range W0–W4 than it was for the fresh colloid, despite the changes in the enhancing medium which occurred over the 4-week storage period. This is a clear demonstration of the effectiveness of the approach of using 2-MI as the IS.

### Extending the Detection Limits of Pano

The data above were obtained with an IS concentration of 2 × 10^–7^ M, which was chosen to be near the low end of the Pano concentration range which was tested. Choosing a low IS concentration is normally desirable since it minimizes competition between the target and IS for surface sites. Indeed, it is essential to have a low concentration if the IS is a strongly binding compound which is preadsorbed to the substrate. In the data shown above, the use of even a low concentration of the IS did slightly increase the lower limit of detection of the Pano from 10^–7^ to 2 × 10^–7^ M.

In principle, it might be expected that the detection range for the target could be tuned by changing the concentration of the IS. Figure 3A shows the log/log calibration curves of Pano for two different concentrations of 2-MI, one similar to that discussed above (line a) and a second which used 10^–5^ M IS (line b). Both plots are linear with *R* ^2^ values of 0.993 and 0.998, respectively and the higher concentration IS allows quantification of Pano at a higher concentrations. However, this result is misleadingly simple, since at intermediate IS concentration the calibration plot was curved (see Figure S7a), also the slope and intercepts of the linear calibrations are different. The linear calibrations fall into two concentration ranges: a low concentration region, where more surface sites than analyte and IS are available, and a high concentration range, where the analyte and IS compete for the limited active surface sites.

**Figure 3:**
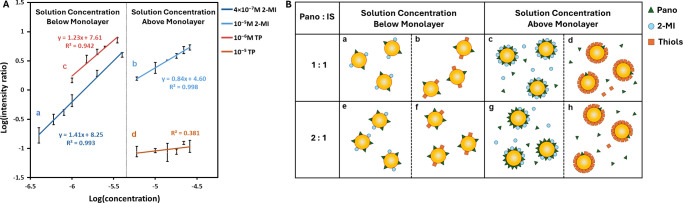
(A) Comparison of calibration curves constructed for the band intensity ratios of Pano and various concentrations of IS (a) 4 × 10^–7^ M and (b) 10^–5^ M 2-MI, (c) 10^–6^ M and (d) 10^–5^ M TP against the Pano concentrations. The values of peak heights and concentrations are logarithmic. (B) Conceptual illustration of the adsorption of mixed components onto the AuNP surface before aggregation. The figure compares the relative amounts of each component on the NP surface at varying Pano to IS (2-MI or TP) ratios and at different total concentrations.

In addition to 2-MI, thiophenol (TP) was tested as an example of a nonchemically matched IS. TP was used because it is a common IS and thiols are well-known for their ability to spontaneously bond to Au and Ag surfaces via strong covalent metal-S bonds;^31,32^ so that TP is expected to have a higher binding constant than Pano.

The same methodology employed in the Pano/2-MI system was used for these investigations, with TP used at two different concentrations. When a low concentration of TP was used as the IS, an increase in the Pano marker bands (1228 cm^–1^) was evident in the spectra with the rising Pano concentration, while the variation in TP marker bands (1020 cm^–1^) was barely discernible (Figure S8a). This is consistent with the high affinity TP IS being completely adsorbed while the remaining surface filled up with the Pano as its concentration was increased. A log/log calibration curve of relative size of the selected fingerprint bands (1228 cm^–1^:1020 cm^–1^) with varying Pano/TP concentration ratios is shown in Figure 3A, line c. This plot is linear (*R* ^2^ = 0.94) with a limit of detection for Pano of 10^–6^ M. This value is higher than was obtained in experiments with 2-MI as the IS (LOD = 4 × 10^–7^ M), presumably due to increased competition for surface sites created by a combination of the higher IS concentration and stronger binding by this IS.

In contrast with the 2-MI case, when a higher concentration of TP (10^–5^ M) was used as the IS, the intensity of the TP band in the mixture was much larger than that of Pano and it continued to dominate the spectra across all concentration ratios, while the Pano marker band remained small over all the concentrations tested (Figure S8b). The log/log calibration curves for 10^–5^ M TP as the IS ([Fig fig3]A, line d) also showed very small changes in the intensity ratio with concentration. This result is broadly due to the fact that Pano could not effectively compete with the higher concentration of thiol IS used in these measurements but there are two cases to consider. First, if the TP concentration is just below that required to completely cover the surface, the calibration will be flat, since the Pano will fill the small number of remaining vacant surface sites at all the concentrations investigated. Second, if the TP concentration is just above monolayer concentration, the Pano and TP will compete for surface sites but the Pano signal will also be small, since the TP has much higher affinity for the surface, although it would still be expected to increase with Pano concentration. It is difficult to discern which of these possibilities applies to the data in Figures 3A and S5b because the low signals result in large error bars and the best fit line an *R* ^2^ value of just 0.38. Regardless of the details, the important point is that these results demonstrate that when TP is used as the IS the detection range for Pano cannot be increased by using a higher concentration of the IS in the same way that was possible for the chemically matched 2-MI. That is not to say that TP cannot perform reasonably well as an IS at low concentrations, which consistent with its widespread use in quantitative SERS measurements.^33,49,50^

Figure 3B brings together diagrams illustrating the various interactions that give rise to the data for both 2-MI and TP shown in Figure 3A. While SERS enhancement primarily arises from hot spots formed during aggregation, we assume that analytes initially adsorbed onto NP surfaces are retained upon aggregation and therefore are automatically present in the enhanced hot spots. For clarity, the figure shows only the first ligand adsorption step. Irrespective of the nature of the IS, there are two broad concentration regions for a system with mixed components adsorbing on metal surfaces. When the number of molecules in the Pano/IS mixture solution is insufficient to completely cover the surface of all the NPs (i.e., the solution concentration is well below the monolayer threshold), there is no competition between Pano and IS molecules, regardless of their respective binding affinities to the metal NPs. Consequently, as illustrated in Figure 3B, the molecules in solution populate the surface of the particles in proportions that reflect the initial mixture ratio. Thus, both 2-MI and TP can effectively function as IS when the total concentration of the mixture is below the monolayer threshold. In both cases, the slope of the signal vs concentration line will simply be determined by the relative SERS intensities of the bands from the Pano and IS which are used to calculate the peak intensity ratio. Conversely, when an excess number of molecules is present in the test solution (i.e., solution concentration above the monolayer threshold), competitive adsorption leads to a formation of self-assembled monolayer (SAM) whose composition is based not just on the relative concentrations of Pano and IS but also on the relative affinities of both to the metal NPs. This difference between submonolayer and high concentration regimes has previously been discussed in the context of modeling surface coverage resulting from coadsorption of mixed thiols.^51,52^ In the high concentration range, molecules which have similar chemical properties will continue to absorb to the surface in a ratio that mirrors their concentration ratio in the mixed sample solution, even at high concentration, as illustrated for the Pano/2-MI system (Figure 3B, panels c and g). Of course, even for a chemically matched IS, the binding affinity may not be identical to that of the target analyte, so the ratio of molecules on the surface will not be exactly follow the concentration ratio since the compound with the higher affinity will be preferentially adsorbed. This can explain why the slopes of the calibration curves for Pano/2-MI are different in the two 2-MI concentration regimes and why the calibration may deviate from linearity at intermediate values as the mechanism changes from one to the other. Specifically, at intermediate IS concentrations (such as 10^–6^ M, as shown in Figure S7a), both independent and competitive adsorption processes likely occur simultaneously and dynamically, leading to a gradual and continuous shift in adsorption behavior. This transition region does not exhibit a simple linear response because the balance between surface site availability and molecular competition changes with concentration.

It is important to note that the ability to obtain quantitative measurements at high target concentration by using high IS concentration is very different from the case where the absolute signal from the sample is used without any IS because in that case the absolute signal from the sample will plateau at high concentrations (as shown in Figure 2B) due to saturation of the surface. This means that the signal changes very little with concentration in the high concentration range. In addition, for a nonchemically matched analyte/IS system, the molecules will have different binding affinities and those with the higher binding affinity (typically the IS) will dominate the surface composition. For example, as shown in Figure 3B, panels d and h, since TP has a higher binding affinity than Pano, at high IS concentration the NPs’ surfaces are mostly covered by TP, regardless of the Pano/TP ratio. These observations highlight the fact that while the detection ranges for a specific chemical target can be extended by altering the concentration of chemically matched IS, the same approach is not possible for nonmatched IS which have different binding affinities.

Given the importance of Pano quantification in the context of TDM, demonstrating the method’s applicability under biologically relevant conditions is valuable. To address potential matrix interference commonly encountered in clinical samples, we introduced 10^–7^ M adenine (Figure S9) as a representative low-molecular-weight metabolite and 0.1% (w/v) albumin (Figure S10) as a representative protein. In both cases of quantitative analysis of Pano, the use of 4 × 10^–7^ M 2-MI as the IS significantly improved linearity (with *R* ^2^ increasing from 0.978 to 0.996 in the presence of adenine, and from 0.952 to 0.971 with albumin) and reduced the standard error of the slope (from 0.114 to 0.035 for adenine and from 0.128 to 0.084 for albumin). Although further optimization of the IS/analyte ratio may enhance performance under varying biological conditions, these results already demonstrate the effectiveness of 2-MI in minimizing matrix effects during quantitative SERS analysis.

## Conclusions

In conclusion, we have used the example of quantitative analysis of Pano to illustrate the potential of a novel approach to identifying IS for SERS measurements by testing small fragments of the large target compound. In this case the fragment which was most suitable as an IS for Pano was 2-MI, since it is chemically similar, and therefore is expected to respond to any perturbations in the experimental conditions in the same way as the target analyte. Use of this chemically matched standard allowed the effect of changes in enhancement factor of an enhancing Au colloid over several weeks to be compensated to the extent that the standard error in the calibration was the similar for fresh material and for measurements carried out over a period of 4 weeks. In addition to compensating for changes in experimental conditions, it was also found that the use of chemically matched IS allowed the calibration range to be extended well above the concentration which completely saturates the enhancing surface. Saturation effects normally limit the quantification range of SERS measurements without IS and here we have shown that it also limits the range if a nonchemically matched strongly absorbing compound, such as the TP, is used as the IS.

Finally, the observation that different calibration curves are obtained at low (submonolayer) and high concentrations has been explained by recognizing that there are two distinct mechanisms governing the adsorption, and therefore the relative intensities of target and IS signals. At low concentrations the target and IS are both free to bind independently, while at high concentrations they compete for a limited number of surface sites. To our knowledge this is first time that these effects have been explicitly discussed for application of IS.

These observations demonstrate the superiority of employing a chemically matched IS in quantitative SERS, which in turn highlights the importance of being able to obtain IS whose properties chemically match those of the target analyte. Previous studies have primarily depended on using isotopologues or compounds which have similar structures to the target to ensure that the IS are chemically matched to the target. We believe that the approach used here, where fragments of the target are rationally selected, will allow wider access to the chemically matched IS approach since it overcomes the availability/cost problems associated with existing methods.

## Supplementary Material



## Notes

UV–vis extinction spectra of as-prepared CRGC and CRGC after aggregation; comparison of the Raman spectrum of Pano with the SERS spectra of Pano on Au and Ag; summary table of the detectability of Pano’s chemical fragments on Au and Ag; chemical structures of reference compounds used for peak assignment of Pano on Au; SERS spectra of Pano and 2-MI on Au showing the bands in the spectra do not change with concentration; SERS spectra for a series of concentrations of Pano detected on Ag with a corresponding calibration plot; quantitative analysis of 2-MI on Au; nonlog/log calibration curves for Pano using 10^–6^ and 10^–5^ M 2-MI as IS; SERS spectra of Pano/10^–6^ and 10^–5^ M TP at various ratios; quantitative analysis of Pano in the presence of both adenine and albumin; and conclusion on the detection ranges of Pano with various types and concentrations of IS (PDF)
