# Supplementary material for: Exploring the Potential of Chemically Matched Fragments as Internal Standards for Quantitative SERS with Panobinostat
Source: Anal Chem. 2025 Aug 21;97(34):18490–8. doi: 10.1021/acs.analchem.5c02017 (PMC12409699; doi:10.1021/acs.analchem.5c02017)
Supplement: Supplementary file 1 [file ac5c02017_si_001.pdf]

Supporting Information

Exploring the Potential of Chemically-Matched Fragments as Internal Standards for Quantitative SERS with Panobinostat

Yiming Huang,[a] Yikai Xu,[b] Chunchun Li,[c] and Steven E. J. Bell[a]*

[a]*School of Chemistry and Chemical Engineering, David Keir Building, Stranmillis Road, Queen's University, Belfast, U.K., BT9 5AG.*

[b]*Key Laboratory for Advanced Materials and Feringa Nobel Prize Scientist Joint Research Center, Frontiers Science Center for Materiobiology and Dynamic Chemistry, School of Chemistry and Molecular Engineering, East China University of Science and Technology, 130 Meilong Road, Shanghai, P. R. China, 200237.*

[c]*School of Materials Science and Engineering, East China University of Science and Technology, 130 Meilong Road, Shanghai 200237, China.*

** Steven E. J. Bell − School of Chemistry and Chemical Engineering, Queen's University Belfast, Belfast, Northern Ireland BT7 1NN, UK; orcid.org/0000-0003-37678985; Email: s.bell@qub.ac.uk.*

Contents

1. UV–vis extinction spectra of as-prepared CRGC and CRGC after aggregation..........................S3
2. Comparison of the Raman spectrum of Pano with the SERS spectra of Pano on Au and Ag S4
3. Summary table of the detectability of Pano's chemical fragments on Au and Ag........................S5
4. Chemical structures of reference compounds used for peak assignment of Pano on Au S6
5. SERS spectra of Pano and 2-MI on Au showing the bands in the spectra do not change with concentration S7
6. SERS spectra for a series of concentrations of Pano detected on Ag with a corresponding calibration plot..S8
7. Quantitative analysis of 2-MI on Au S9
8. Non-log/log calibration curves for Pano using 10^{-6} M and 10^{-5} M 2-MI as IS...........................S10
9. SERS spectra of Pano/10^{-6} M and 10^{-5} M TP at various ratios S11
10. Quantitative analysis of Pano in the presence of adenine S12
11. Quantitative analysis of Pano in the presence of albumin..S13
12. Conclusion on the detection ranges of Pano with various types and concentrations of IS S14

1. UV–vis extinction spectra of as-prepared CRGC and CRGC after aggregation

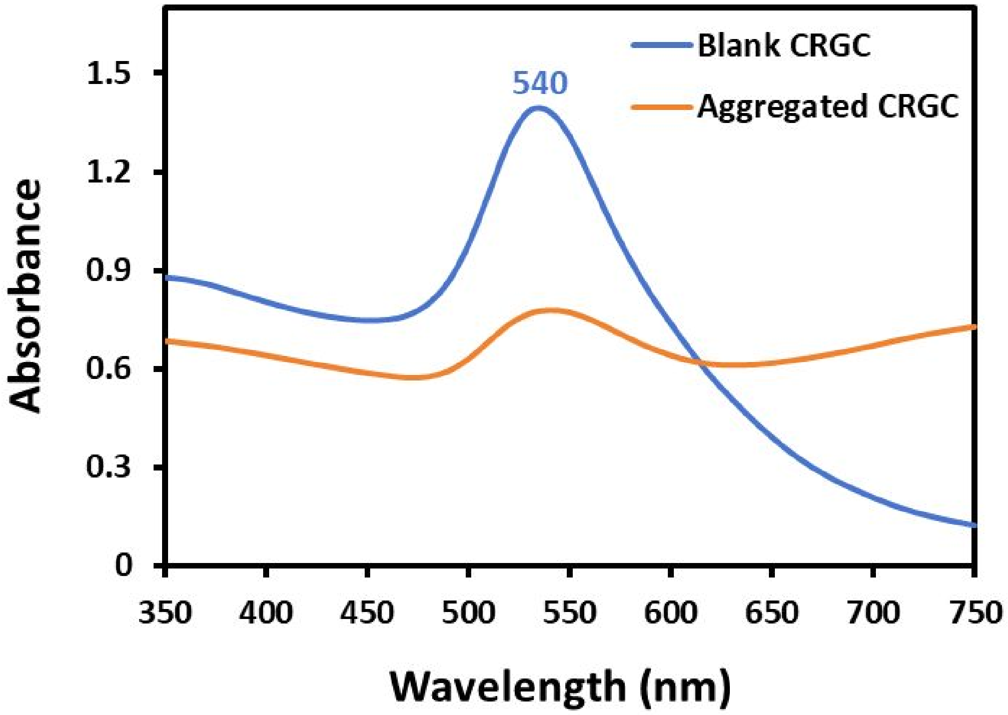

Figure S1. UV–vis extinction spectra of as-prepared CRGC (blue line) and CRGC after aggregation with $(NH_4)_2SO_4$ (orange line). The unaggregated CRGC exhibits a single extinction peak around 540 nm, while the aggregated CRGC shows peak broadening and decreased intensity, indicating the formation of larger NP aggregates.

2. Comparison of the Raman spectrum of Pano with the SERS spectra of Pano on Au and Ag

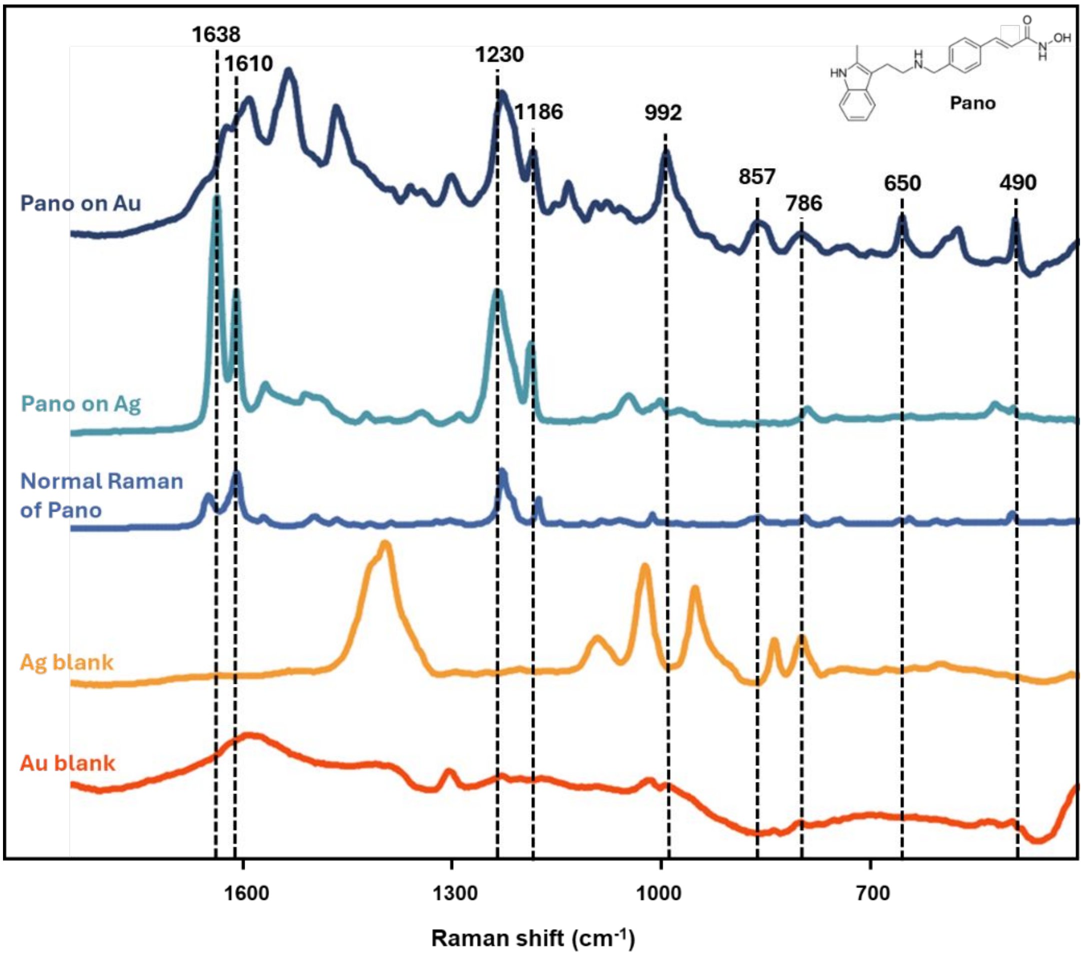

Figure S2. Normal Raman spectra of solid Pano and SERS spectra of final concentration 10^{-5} M of Pano on Au and Ag (upper three lines); SERS spectra of aggregated CRSC and CRGC (lower two lines). Spectra are vertically offset for clarity.

3. Summary table of the detectability of Pano's chemical fragments on Au and Ag

	CRGC	CRSC
2-Methylindole	✓	×
4-Methylbenzylamine	✓	×
Acrylamide	×	×
Tryptamine	✓	✓
Acetohydroxamic acid	×	✓
Pyrrole	✓	×

Table S1. The list of fragments of Pano detected showing the result of detectability of CRGC and CRSC with each fragment.

4. Chemical structures of reference compounds used for peak assignment of Pano on Au

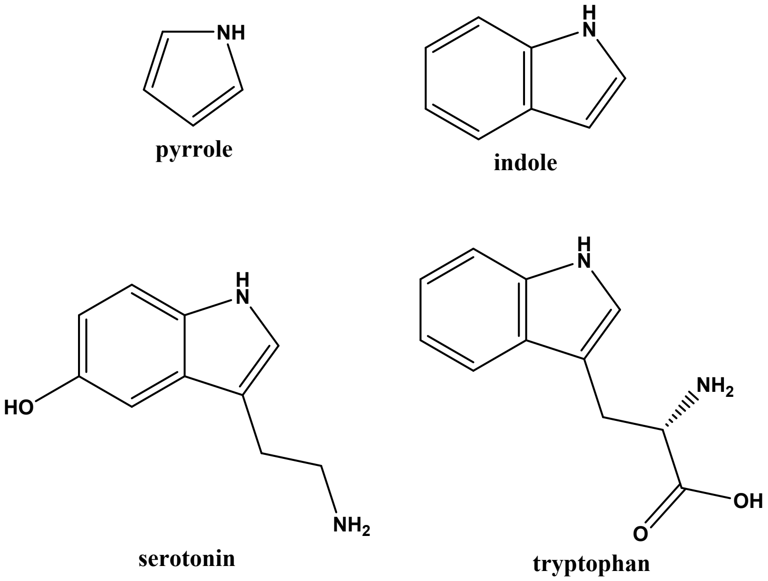

Figure S3. Chemical structures of pyrrole, indole, serotonin, and tryptophan, which were used as reference compounds for peak assignment in the analysis of Pano.

5. SERS spectra of Pano and 2-MI on Au showing the bands in the spectra do not change with concentration

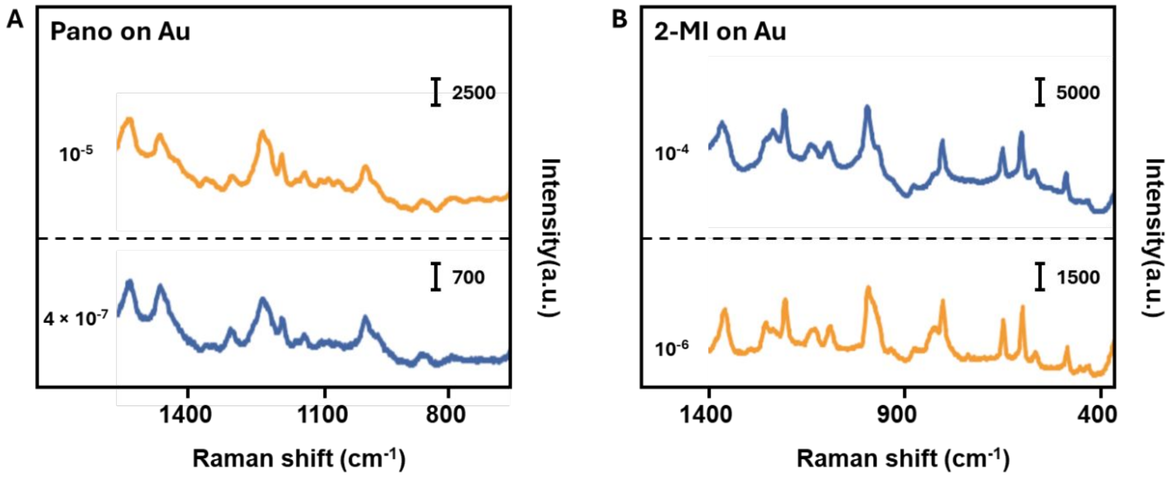

Figure S4. SERS spectra of Pano and 2-MI on Au at the concentrations marked in mol dm^{-3} showing the bands in the spectra do not change with concentration.

6. SERS spectra for a series of concentrations of Pano detected on Ag with a corresponding calibration plot

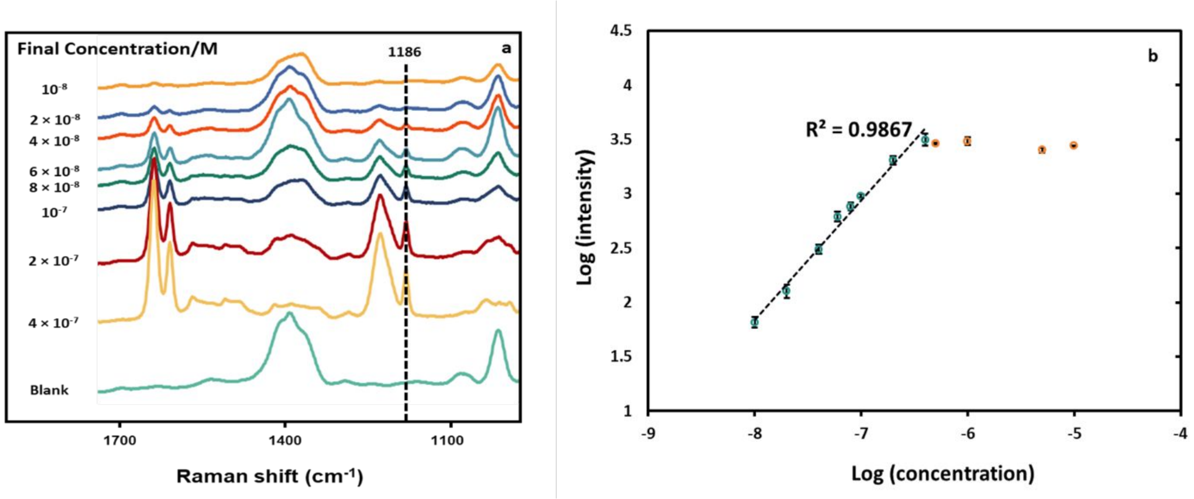

Figure S5. (a) SERS spectra of a series of concentrations (indicated with each line in figure) of Pano detected on Ag. Targeted characteristic band at 1186 cm^{-1} is marked. Spectra are vertically offset for clarity. (b) Corresponding calibration curve for SERS signal intensity (1186 cm^{-1}) versus Pano concentration on Ag. The values of concentration and intensity in the plot are logarithmic.

7. Quantitative analysis of 2-MI on Au

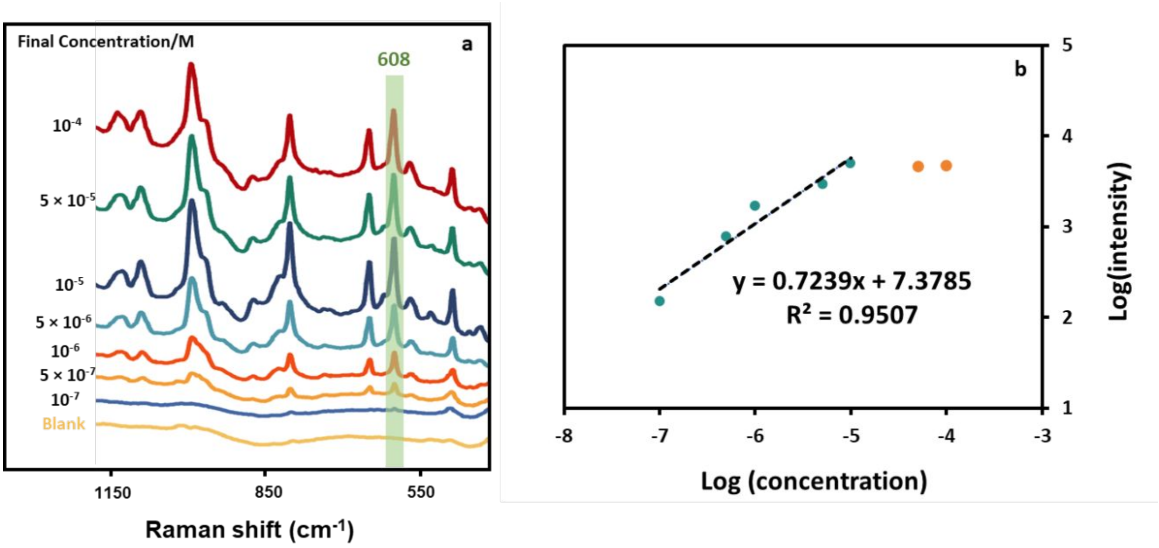

Figure S6. (a) SERS spectra of a series of concentrations (indicated with each line in figure) of 2-MI detected on Au. Targeted characteristic band at 608 cm^{-1} is marked. Spectra are vertically offset for clarity. (b) Corresponding calibration curve for SERS signal intensity (608 cm^{-1}) versus 2-MI concentration on Au. The values of concentration and intensity in the plot are logarithmic.

8. Non-log/log calibration curves for Pano using 10^{-6} M and 10^{-5} M 2-MI as IS

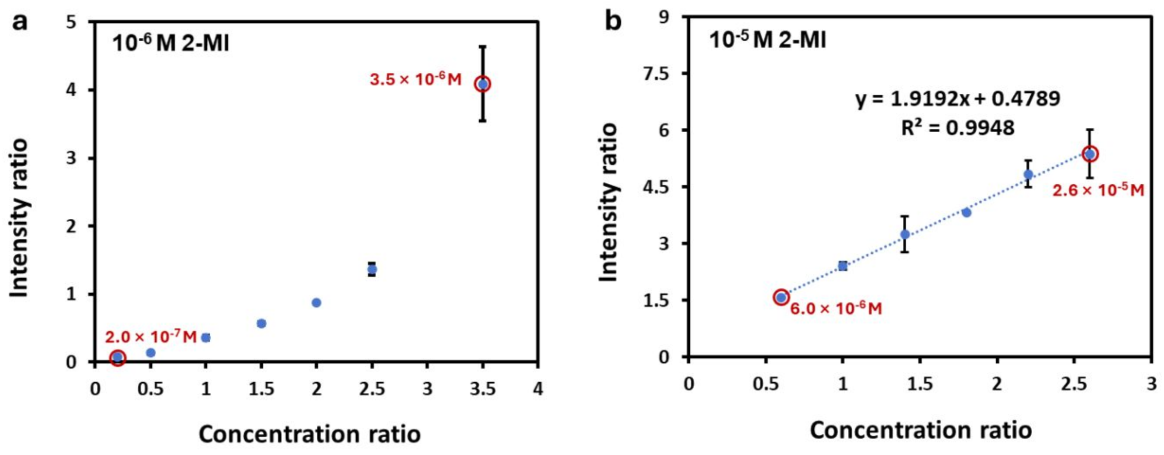

Figure S7. Calibration curves of Pano/2-MI concentration ratio against Pano/2-MI peaks intensity ratio (1186:608 cm^{-1}) on Au with different concentrations of 2-MI as IS: 10^{-6} M and 10^{-5} M. The concentrations of Pano corresponding to the boundary ratio points in each plot are labelled accordingly.

9. SERS spectra of Pano/10^{-6} M and 10^{-5} M TP at various ratios

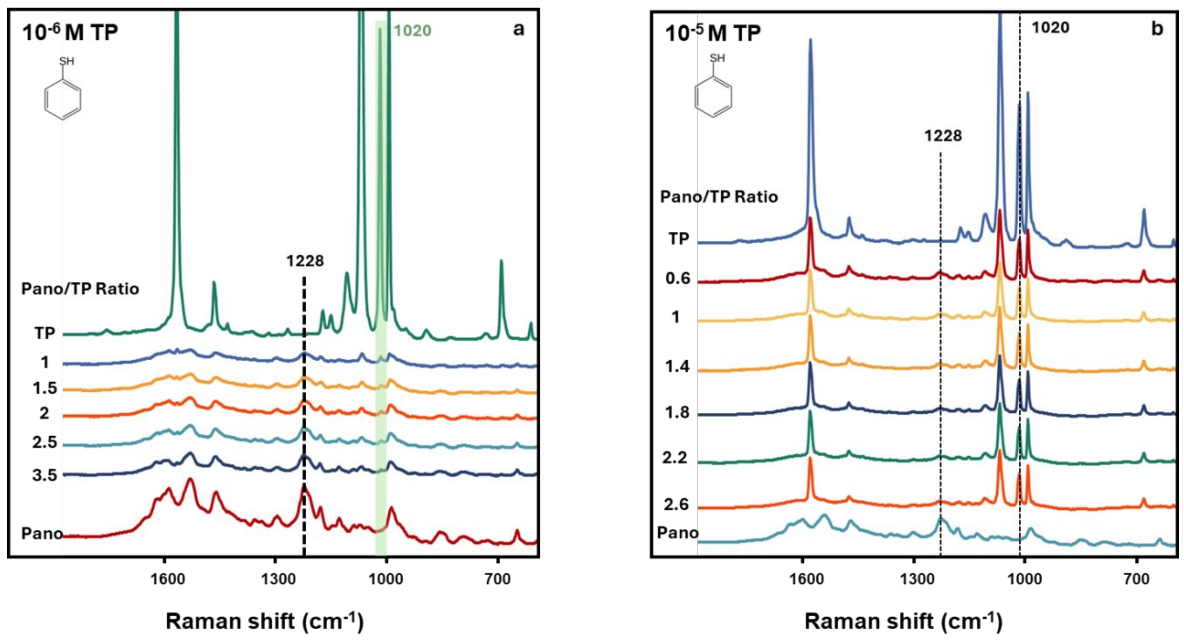

Figure S8. SERS spectra of Pano/TP mixtures at various ratios (labelled with each line) detected on Au with 10^{-6} M TP (a) and 10^{-5} M TP (b). Characteristic band for Pano (1228 cm^{-1}) and TP (1020 cm^{-1}) are marked. Spectra are vertically offset for clarity.

10. Quantitative analysis of Pano in the presence of adenine

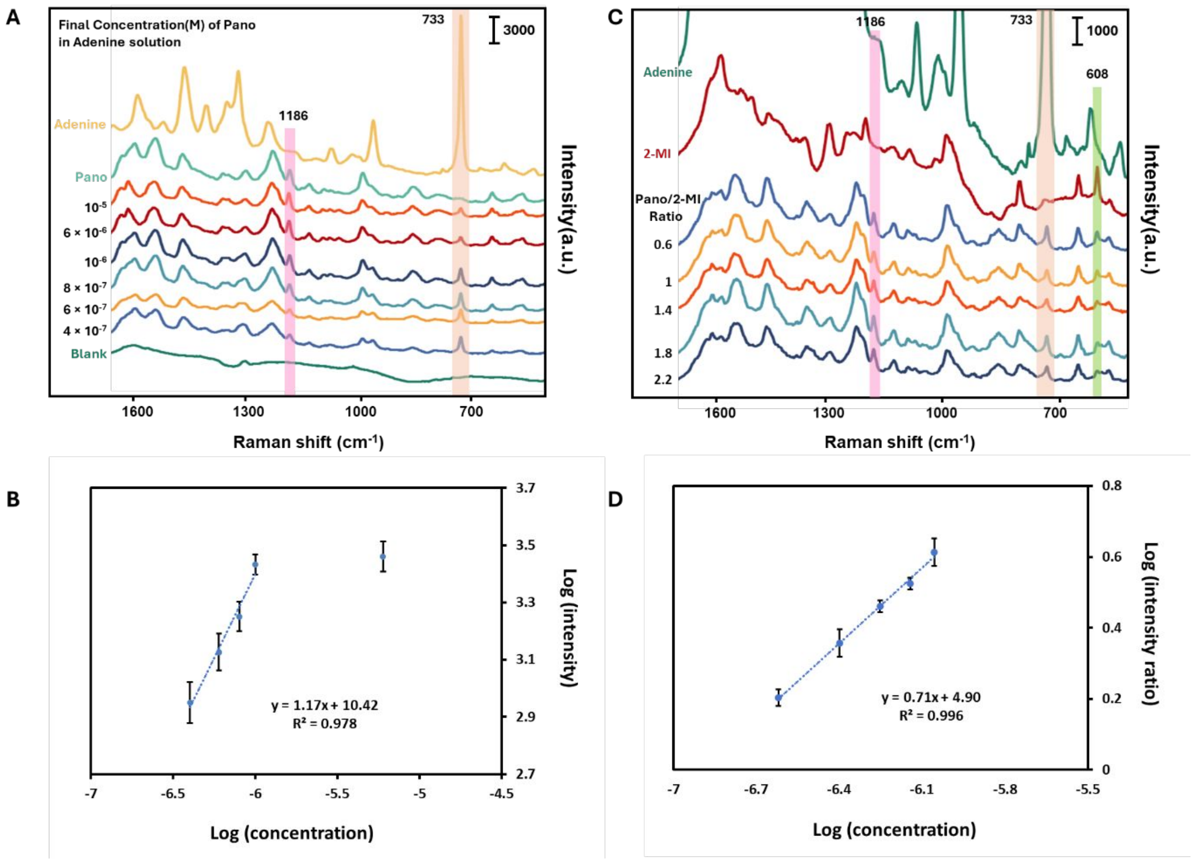

Figure S9. (A) SERS spectra of a series of Pano concentrations (marked on each trace in the figure) recorded on Au in the presence of 10^{-7} M adenine. The band at 1186 cm^{-1}, used for calibration, and the characteristic adenine peak at 733 cm^{-1} are highlighted. (B) Logarithmic calibration curve for SERS signal intensity (1186 cm^{-1}) versus Pano concentration on Au in the presence of adenine. (C) SERS spectra of a series of Pano/2-MI mixtures with different ratios detected on Au in the presence of 10^{-7} M adenine. Characteristic bands for Pano (1186 cm^{-1}), 2-MI (608 cm^{-1}) and adenine (733 cm^{-1}) are marked. (D) Plot of the logarithmic intensity ratio of Pano/2-MI characteristic bands (1186:608 cm^{-1}) on Au against the logarithmic concentration of Pano in the presence of adenine. Error bars for the data are calculated from 3 replicate experiments. The concentration of 2-MI used was 4×10^{-7} M.

11. Quantitative analysis of Pano in the presence of albumin

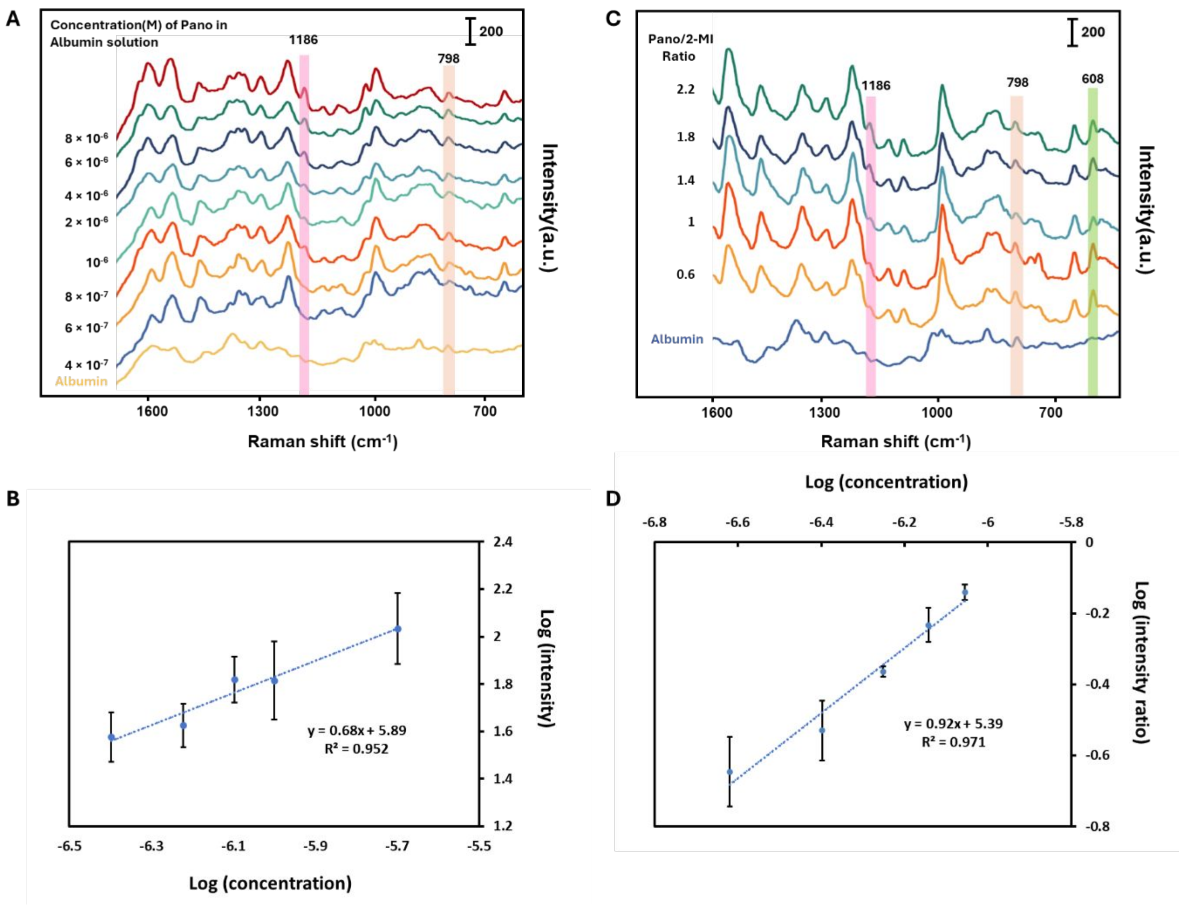

Figure S10. (A) SERS spectra of a series of Pano concentrations (as indicated for each trace in the figure) recorded on Au in the presence of 0.1% (w/v) albumin. The band at 1186 cm^{-1}, used for calibration, and the potential characteristic albumin peak at 798 cm^{-1} are highlighted. (B) Logarithmic calibration curve for SERS signal intensity (1186 cm^{-1}) versus Pano concentration on Au in the presence of albumin. (C) SERS spectra of a series of Pano/2-MI mixtures with different ratios detected on Au in the presence of 0.1% (w/v) albumin. Characteristic band for Pano (1186 cm^{-1}), 2-MI (608 cm^{-1}) and albumin (798 cm^{-1}) are marked. (D) Plot of the logarithmic concentration of Pano against the logarithmic intensity ratio of Pano/2-MI characteristic bands (1186:608 cm^{-1}) on Au in the presence of albumin. Error bars for the data are calculated from 3 replicate experiments. The concentration of 2-MI used was 4×10^{-7} M.

12. Conclusion on the detection ranges of Pano with various types and concentrations of IS

IS	Concentration of IS	Lower limit of Pano	Upper limit of Pano
Without		10^{-7} M	2×10^{-6} M
2-MI	2×10^{-7} M	10^{-7} M	2.2×10^{-6} M
	4×10^{-7} M	4×10^{-7} M	4×10^{-6} M
	10^{-6} M	2×10^{-7} M	3.5×10^{-6} M
	10^{-5} M	2×10^{-6} M	2.6×10^{-5} M
TP	10^{-6} M	10^{-6} M	3.5×10^{-6} M
	10^{-5} M	10^{-6} M	6×10^{-6} M
BZM	10^{-5} M	10^{-6} M	6×10^{-6} M

Table S2. The limits of detection for Pano on Au determined using various concentrations of chemically-matched IS, 2-MI, as well as other chemically-different ISs, TP and benzyl mercaptan (BZM); and the detection limits assessed without the presence of IS (single drug).
